# Effects of deer on the photosynthetic performance of invasive and native forest herbs

**DOI:** 10.1093/aobpla/plx011

**Published:** 2017-03-20

**Authors:** J. Mason Heberling, Nathan L. Brouwer, Susan Kalisz

**Affiliations:** 1 Department of Ecology and Evolutionary Biology, University of Tennessee, 569 Dabney Hall, Knoxville, TN 37996, USA; 2 Section of Botany, Carnegie Museum of Natural History, 4400 Forbes Avenue, Pittsburgh, PA 15213, USA; 3 Department of Conservation and Field Research, National Aviary, Allegheny Commons West, 700 Arch Street, Pittsburgh, PA 15212, USA

**Keywords:** *Alliaria petiolata*, biological invasion, deer facilitation, ecophysiology, functional traits, light availability, *Maianthemum racemosum*, *Trillium grandiflorum*

## Abstract

Overabundant generalist herbivores can facilitate non-native plant invasions, presumably through direct and indirect modifications to the environment that affect plant performance. However, ecophysiological mechanisms behind ungulate-mediated plant invasions have not been well-studied. At a long-term *Odocoileus virginianus* (white-tailed deer) exclusion site in a temperate deciduous forest, we quantified deer-mediated ecophysiological impacts on an invasive biennial *Alliaria petiolata* (garlic mustard) and two palatable native herbaceous perennials, *Maianthemum racemosum* and *Trillium grandiflorum*. In mid-summer, we found that leaf-level light availability was higher in unfenced areas compared with areas fenced to exclude deer. *Alliaria* in unfenced areas exhibited 50 % higher mean maximum photosynthetic rates compared with fenced areas. Further, specific leaf area decreased by 48 % on average in unfenced areas, suggesting leaf structural responses to higher light levels. Similarly, *Maianthemum* had 42 % higher mean photosynthetic rates and 33 % decreased mean specific leaf area in unfenced areas, but these functional advantages were likely countered by high rates of deer herbivory. By contrast, *Trillium* exhibited significantly lower (26 %) maximum photosynthetic rates in unfenced areas, but SLA did not differ. Deer-mediated differences in light saturated photosynthetic rates for all three species were only significant during months with overstory tree canopy cover, when light availability in the herb layer was significantly lower in fenced areas. *Alliaria*’s enhanced photosynthetic rates implicate overabundant deer, a situation that is nearly ubiquitous across its invaded range. Collectively, our results provide empirical evidence that generalist herbivores can alter non-native plant physiology to facilitate invasion.

## Teaser

The notion that deer can facilitate plant invasions has been increasingly appreciated but the mechanisms remain unclear. In a deciduous forest stressed by overabundant deer and plant invasions, midsummer understory light levels were lower in fenced areas where deer were excluded. Further, invasive garlic mustard (Alliaria petiolata) exhibited 50% higher maximum photosynthetic rates in areas where deer were present. This trend was similar in one native (Maianthemum racemosum) while another native (Trillium grandiflorum) showed the opposite pattern. These results indicate that deer can affect the ecophysiology of invasive species indirectly through modifications to the understory.

## Introduction

The globalization of human activities has led to widespread movement of plants around the world (van Kleunen *et al.* 2015) and species invasions are now widely appreciated as a major source of environmental change (Vitousek *et al.* 1997). In efforts to mechanistically understand, predict, and prevent invasions in an era of ecological change, recent research on plant invasions has focused on functional traits of non-native species that may make them invasive (e.g. van Kleunen *et al.* 2010) and characteristics of communities that may make them susceptible to invasion (Rejmanek *et al.* 2013). Habitats characterized by frequent disturbance or altered disturbance regimes have long been considered more susceptible to plant invasions (e.g. Elton 1958; Hobbs and Huenneke 1992). Many invaders come from regions with a long history of human disturbance (e.g. Fridley 2008) and exhibit specific trait adaptations that may allow them to capitalize on high resource availabilities in disturbed environments (Alpert *et al.* 2000; Davis *et al.* 2000; Daehler 2003). In addition to altering resource availability, human-mediated disturbances can reduce competition from natives that facilitates invasion by disturbance-adapted species (Daehler 2003).

Untangling the historical, environmental and ecological factors that dictate plant invasion success in disturbed environments remain a significant research challenge, especially for ecosystems stressed by several potentially interacting factors (Dávalos et al. 2015a). Temperate forest ecosystems have been subjected to multiple biotic stressors worldwide, including historically unprecedented increases in ungulates (e.g. deer, elk, moose, goats, feral pigs; Singer *et al.* 1984; Wardle *et al.* 2001; Côté et al. 2002). In eastern North America, white-tailed deer (*Odocoileus virginianus*) densities have risen far above what has been considered to be compatible with forest health and regeneration (e.g. Diefenbach *et al.* 1997), driving further ecological change and associated conservation concerns (McShea 2012). For example, deer can have direct consumptive effects through selective foraging (e.g. Wiegman and Waller 2006; Mason *et al.* 2010; Averill *et al.* 2016), indirect effects on biotic and abiotic properties (Rooney and Waller 2003; Heckel *et al.* 2010; Kardol *et al.* 2014; Heckel and Kalisz 2017) and can increase exotic seed dispersal (Myers *et al.* 2004). Together, these effects have resulted in significant and negative community-level impacts (see Habeck and Schultz 2015 for a meta-analysis). Although traditionally studied as separate phenomena, a growing literature has linked deer overabundance to increases in fitness and abundance of invasive plant species (Vavra *et al.* 2007; Eschtruth and Battles 2009; Knight *et al.* 2009; Christopher *et al.* 2014; Kalisz *et al.* 2014; Dávalos *et al.* 2015*a*; Shen *et al.* 2016).

In a temperate deciduous forest in North America, we investigated the effects of overabundant deer on the ecophysiological performance of invasive *Alliaria petiolata* (Brassicaceae, garlic mustard; hereafter *Alliaria*) and two commonly co-occurring native species preferred by deer as forage, *Trillium grandiflorum* and *Maianthemum racemosum*. Kalisz *et al.* (2014) recently reported strong population-level demographic declines in *Alliaria* at this site where deer were excluded. Declines were so strong that in the absence of deer, population growth rates fell below replacement (*λ* <1), which if maintained, projects eventual local extinction of this invader where deer are excluded. Comparable trends in abundance have been reported for additional invaders in other deciduous forests (Eschtruth and Battles 2009; Dávalos *et al.*2015*a*; Shen *et al.* 2016). While other studies have investigated potential physiological impacts of *Alliaria* on native species (Hale *et al.* 2011, 2016) and *Alliaria* photosynthetic physiology in general (Dhillion and Anderson 1999; Anderson and Cipollini 2013; Stinson and Seidler 2014), the physiological basis for deer-mediated plant invasions remains unexplained.

We hypothesize that persistent browsing by overabundant deer on palatable herbaceous species (Knight *et al.* 2009) significantly increases light availability, which favours forest edge- or gap-adapted species like *Alliaria*. To assess this hypothesis, we measured *in situ* leaf-level ecophysiology of *Alliaria* and two native perennials, including metrics of leaf photosynthetic performance and specific leaf area. Where deer were allowed access (unfenced areas), we predicted increased photosynthetic rates for *Alliaria* due to deer-induced disturbances that increase light availability (deer facilitation). In contrast, we predicted the opposite pattern for the palatable natives due to high rates of deer herbivory (which can exceed 75 % of flowering stems; Kalisz *et al.* 2014) and negative non-consumptive effects (Heckel *et al.* 2010) that likely constrain their physiological performance. Further, as native understory species are presumably well-adapted to low light levels in the forest interior, we hypothesized that any increases in light availability would be more beneficial to the more light-demanding *Alliaria* than relatively shade tolerant natives. 

## Methods

### Study site and focal species

The study was conducted in a closed canopy deciduous forest in south western Pennsylvania, USA (Trillium Trail Nature Reserve: 40.5201°N; 79.9010°W). Trillium Trail is a 16 ha tract of forest within >125 ha of forest and parkland in Pittsburgh, PA. It has a diverse herbaceous understory that has been managed by the Borough of Fox Chapel since 1949. Dominant overstory species include *Acer saccharum* (sugar maple) and *Fagus grandifolia* (American beech), as well as *Fraxinus* spp. (ash), *Carya* spp. (hickory), *Quercus alba* and *Q. rubra* (white and red oak), *Prunus serotina* (black cherry) and *Liriodendron tulipifera* (tulip poplar).

Trillium Trail provides an appropriate site for testing ecological hypotheses on deer, plant invasions and their interactions for several reasons. Deer densities at the site rapidly increased in the early 1990s, resulting in annual browse rates on palatable herb species rising as high as 100 %, especially for flowering individuals (Knight *et al.* 2009). Winter aerial flyovers from 1993 to 2004 confirm high deer densities at an estimated 20–42 deer per km^2^ compared with historic estimates of 10–12 deer per km^2^ (Kalisz *et al.* 2014). In addition, the diverse understory is being actively invaded by several woody and herbaceous exotic species. In particular, the non-native biennial herb *A.petiolata* is now common throughout the forest understory, but this invasion was relatively recent, as *Alliaria* was reported as rare or nearly absent before 1990 (Knight *et al.* 2009).

In 1999, the Borough of Fox Chapel erected a 2.5 m tall fence around a 10.06 ha portion of the forest in an effort to protect the understory diversity of Trillium Trail from deer browse (see Knight *et al.* 2009). Fencing (15 cm × 15 cm steel mesh) excluded deer, but allowed other animals free access. These fenced and unfenced areas are in the same forest and have similar vegetation to the long-term demographic plots in Kalisz *et al.* (2014). To avoid damage to the long-term demographic plots, we only studied plants inside and outside of the adjacent larger fenced area. Therefore, our results can be compared with demographic results in Kalisz *et al.* (2014), but we did not measure the same individuals.

To compare the effects of deer on *Alliaria* to those of resident species, we also studied two native perennial herbs that commonly co-occur with *Alliaria* at the site, *M.racemosum* (Asparagaceae, false Solomon’s seal) and *T.grandiflorum* (Melanthiaceae, white/large-flowered trillium). Other *Trillium* species are found in adjacent areas of the forest (e.g. *T. erectum* var. *album*) and non-flowering individuals are difficult to distinguish. However, *T. grandiflorum* is the dominant *Trillium* species in the areas of the forest we sampled. As such, we assume that all individuals we measured were *T. grandiflorum*. 

### Leaf gas exchange

We measured individuals of the three species from late July 2014 to June 2016, from both fenced and unfenced areas. Individuals were selected haphazardly each day, alternating the daily order that species were measured to prevent any bias by time of day. This approach maximized sample size while permitting inferences on population trait means through the year. For each species, all measured individuals were separated by at least 2 m. For *Trillium* and *Maianthemum*, only adults were measured. For *Alliaria*, 1st year rosettes (i.e. not bolting) were measured in July–March, and 2nd year adults (i.e. bolting/flowering) were measured in April–June (senescence).

Gas exchange measurements were performed *in situ* on intact mature leaves using two LI-6400XT portable photosynthesis systems equipped with a CO_2_ control module, 2 cm × 3 cm leaf cuvette and a red-blue LED light source (LI-COR, Lincoln, NE, USA). All leaves filled the leaf cuvette entirely. Measurements were made under ambient temperature and humidity, with reference chamber CO_2_ concentration at 400 μmol mol^−1^. Leaves were photo-induced at a moderate irradiance level (200–400 μmol photons m^−2^ s^−1^) until they equilibrated. Light levels were then progressively increased until leaves were light saturated (800–1500 μmol photons m^−2^ s^−1^) prior to employing the measurement routines described below. All individuals were light saturated at the highest light levels, with no apparent signs of photoinhibition. 

#### Seasonal measurements

For each species, light-saturated photosynthetic rate (*A_sat_*) was recorded after equilibration to the measurement light level for at least 2 min at high photosynthetic photon flux density (PPFD; 800–1500 μmol photons m^−2^ s^−1^) and reaching defined stability parameters based on photosynthetic rate and stomatal conductance. Measurements were taken at least once per month, under ambient leaf temperature and humidity. As a biennial with green leaves throughout the winter, *Alliaria* was measured in all months except February and March (due to snow cover and low temperatures). The native perennials (both deciduous) were measured from leaf expansion in spring until leaf senescence (*Maianthemum*: May–August; *Trillium*: April–June). Cumulatively, these gas exchange measurements resulted in >2800 logged observations over 37 days: 240 total *Alliaria* individuals (mean number of individuals measured per month ± 1 SE: 22 ± 4), 125 *Maianthemum* (29 ± 8) and 79 *Trillium* (11 ± 6).

#### Midseason light and CO_2_ response curves

We measured leaf photosynthetic responses to irradiance (PPFD) in 10–12 steps from 0 to 1500 μmol photons m^−2^ s^−1^. Net photosynthetic rate was recorded after allowing the leaf area inside the cuvette to equilibrate for at least 2 min at each PPFD and achieving defined stability parameters described above. Leaf temperature was maintained at 25 °C under ambient humidity.

Light response curve parameters were estimated through non-linear least squares regression of a non-rectangular hyperbola (Marshall and Biscoe 1980) **[see Supporting Information—File S1]**:

$$A_{net}=\frac{\varphi PPFD+A_{max}-\sqrt{{\left(\varphi PPFD+A_{max}\right)}^{2}-4\theta \varphi PPFD\left(A_{max}\right)}}{2\theta }-R_{d}$$

where *A_net_* and *A_max_* are the area-based net and maximum gross photosynthetic rates (μmol CO_2_ m^−2^ s^−1^), respectively, *φ* is the apparent quantum yield (mol CO_2_ mol photons^−1^), *R_d_* is daytime dark respiration rate (|*A_net_*| at no light; μmol CO_2_ m^−2^ s^−1^) and *θ* is curve convexity (dimensionless). Light compensation point (LCP) was calculated as the *x*-axis intercept, and light saturation point (LSP) as the PPFD when 90 % of *A_max_* (model asymptote) was achieved.

After light response curve measurement, leaves were re-stabilized at the saturated light level to measure photosynthetic responses to changing intercellular CO_2_ pressure (photosynthetic rate vs. intercellular CO_2_ partial pressure; *A*/*C_i_* curves). *A*/*C_i_* measurements were taken from the same leaves (or from adjacent nodes) as those for light response curves. While maintaining saturating light levels (800 μmol photons m^−2^ s^−1^), reference chamber CO_2_ concentration was varied in 11 steps (400, 300, 200, 100, 50, 400, 400, 600, 800, 1000, 1500  μmol CO_2_ mol^−1^). Net photosynthetic rates were recorded at each step following equilibration, as described above.

The biochemical photosynthetic model developed by Farquhar *et al.* (1980) was fit using non-linear least squares regression and the simultaneous estimation method described by Dubois *et al* (2007):

$$A_{net}=min\left(A_{v},A_{j}\right)-R_{day}$$

where *A_net_* is the net photosynthetic rate, *A_v_* and *A_j_* are the CO_2_ assimilation rates limited by RuBisCO (Ribulose-1,5-bisphosphate carboxylase/oxygenase; carboxylation) and RuBP (Ribulose-1,5-bisphosphate; substrate regeneration), respectively, and *R_day_* is the daytime mitochondrial respiration rate.

RuBP-saturated (CO_2_ limited) photosynthesis was modelled as:

Av= Vc,maxCi-Γ*Ci+Kc(1+OKO)

where *C_i_* is intercellular CO_2_ partial pressure (Pa), *K_c_* and *K*_0_ are Michaelis–Menten constants for carboxylation and oxygenation (40.4 Pa and 24.8 kPa, respectively), *O* is the O_2_ concentration (21 kPa), *V_c_*_,__*max*_ is maximum carboxylation rate (μmol CO_2_ m^−2^ s^−1^), *R_day_* is the daytime mitochondrial respiration rate (μmol CO_2_ m^−2^ s^−1^) and Γ* is the CO_2_ compensation point in the absence of mitochondrial respiration (3.7 Pa). RuBisCO kinetic constants were obtained from von Caemmerer (2000).

RuBP-limited photosynthesis was modelled as:

$$A_{j}=\;J_{max}\frac{C_{i}-\Gamma^{*}}{{4C}_{i}+8\Gamma^{*}}$$

where *J_max_* is the light saturated maximum rate of photosynthetic electron transport.

### Specific leaf area

On 29 June 2015 (*Alliaria* and *Maianthemum*) and 22 June 2016 (*Trillium*), mature leaves of 19–100 individuals of each species (*Alliaria: n* = 50 1st year rosettes, 50 adults*; Maianthemum*, *n* = 50; *Trillium*, *n* = 19) were sampled by walking three parallel transects and haphazardly collecting 2–5 leaves per individual and ensuring individuals of each species were separated by at least 2 m. Entire leaves were sampled except for *Trillium*, where leaf samples of fixed area (5.32 cm^2^) were taken. Fresh leaves were scanned and leaf area determined using ImageJ (Schneider *et al.* 2012). Leaves were oven dried at 60 °C for at least 48 h. Specific leaf area (cm^2^ g^−1^) was calculated as the leaf surface area per g dry mass.

### Understory light availability

To determine if light levels were different inside versus outside of the fenced area, we used light measurements (PPFD) taken at the height of the individual plants we studied (5–60 cm) that were recorded concurrently with gas exchange measurements with an external LI-COR quantum sensor on the chamber head.

### Data analysis

All statistical analyses were performed in R (R Core Team 2015)**[see Supporting Information—Tables S1–S3 for trait data]**. When necessary, data were log-transformed to meet normality and homoscedasticity assumptions for model residuals.

#### Comparing effects of fencing on leaf traits

For univariate traits, we fit linear mixed-effect models separately for each species (Bates *et al.* 2011). In models for midseason photosynthetic and SLA data, individual and measurement date were random effects. For light response and CO_2_ response curve parameter fits, values were weighted by the precision of the estimates (standard error^−1^). We analysed *A_sat_* data using separate ANOVAs for each species with fixed factors of fencing (fenced or unfenced) and month. We tested for differences within each month with *t*-tests implemented through the multcomp package in R (Hothorn *et al.* 2008).

#### Determining canopy closure

To quantify canopy closure, we measured understory PPFD with a quantum sensor (Onset Computer Corporation, MA, USA) at a fixed location within the fenced deer exclusion area **[see Supporting Information—Fig. S1]**. We then modelled mean daily PPFD (10:00–14:00 h) from April–July using a generalized additive model (GAM), implemented through the mgcv package in R (Wood 2016). After complete canopy closure, light levels are likely to vary little between days. We calculated the derivative of the GAM regression line and its 95 % confidence interval to estimate the first day when the slope of the regression was not significantly different from zero, indicating complete overstory canopy closure **[see Supporting Information—Fig. S2]**.

#### Modelling impact of deer exclusion on understory light availability

For each plant, we calculated mean PPFD conditions experienced while the gas exchange data were collected. On the basis of our analysis of the process of canopy closure (described above), we used data collected between mid-May and late June to determine how deer exclusion affected light conditions before and after complete canopy closure. We modelled log(mean PPFD) as a function of day of year (continuous), fencing (unfenced vs. fenced) and a (day of year) × (fencing) interaction using the lme function in the nlme package in R (Pinheiro *et al.* 2016). Day was included as a random effect. Estimated variances were allowed to change as the growing season progressed and also between fenced and unfenced treatments using the varPower function in lme. We tested the significance of model terms using likelihood ratio tests of nested models. The best model was re-fit without the log transformation to report estimates on the original scale.

## Results

### Understory light availability and deer exclusion

**Figure 1:**
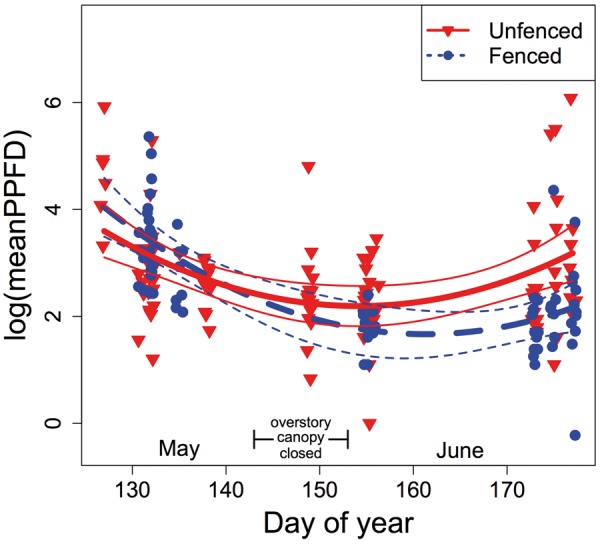
Leaf-level photosynthetic photon flux density (PPFD; μmol photons m^−2^ s^−1^) in fenced and unfenced areas during May and June 2015. Points denote the log transformed plant-level means of 1–16 PPFD measurements (mean = 5.95) for 75 fenced plants (red triangles, solid line) and 110 unfenced plants (blue circles, dashed line) on 11 different days. Regression lines show predictions from a linear mixed model. Error bands represent 95 % confidence intervals. Estimated period for complete overstory canopy closure is denoted (day 143–153; see Section Methods).

At the onset of overstory canopy closure, leaf-level PPFD was not significantly different between fenced and unfenced areas (estimated mid-May unfenced mean PPFD ± 1 SE = 50.8 ± 12.5 μmol photons m^−2^ s^−1^; fenced: 57.4 ± 7.7 μmol photons m^−2^ s^−1^). PPFD decreased significantly as canopy closure progressed through May and June (Table 1; **[see Supporting Information—Figs S1, S2]**) but the pattern of change was significantly different between fenced and unfenced areas (time × fencing: *P* < 0.001, Table 1, Fig. 1). While mean leaf-level PPFD did not change much after mid-May where deer had access, it was ∼70 % lower where deer were excluded (estimate late June mean PPFD unfenced = 52.03, SE = 11.27 μmol photons m^−2^ s^−1^; mean fenced = 15.63, SE = 4.95 μmol photons m^−2^ s^−1^).

**Table 1.** Results of linear mixed model to test for divergence in light availability (leaf-level photosynthetic photon flux density; PPFD) between unfenced and fenced areas (fencing). Data were collected on 11 separate days in May–June 2015 to capture the period of canopy closure. *N* = 75 fenced plants, 110 unfenced plants. Response variable was the mean PPFD of 1–16 measurement per plant (median = 3, mean = 5.95, mode = 3).

PPFD as a function of:	df	Likelihood Ratio	*P*
Time	(5,6)	5.33	0.021*
Time^2^	(6,7)	10.56	0.001**
Fencing	(7,8)	3.76	0.052
Time × fencing	(8,9)	15.67	<0.001***

*P<0.05; **P<0.01; ***P<0.001

### Functional comparisons between fenced and unfenced areas

#### Seasonal measurements

**Figure 2:**
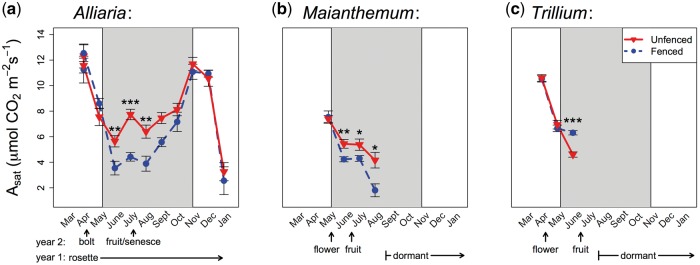
Light saturated photosynthetic rates (*A_sat_*) for (a) *Alliaria petiolata*, (b) *Maianthemum racemosum* and (c) *Trillium grandiflorum* in unfenced (red triangles, solid line) and fenced (blue circles, dashed line) areas. Points denote monthly mean* *±* *1 SE. Grey region illustrates approximate period of overstory canopy shading. Asterisks indicate statistical significance for differences between fenced and unfenced by month: *P <* *0.05; **P <* *0.01; ***P <* *0.001.

*Alliaria’s* highest photosynthetic rates (*A_sat_*) were during periods of high light in the early spring (March, April) and fall (November), when the perennial natives, *Trillium* and *Maianthemum*, were dormant (Fig. 2). All three species had peak light-saturated photosynthetic rates (*A_sat_*) in the spring, which substantially declined following overstory canopy closure.

**Figure 3:**
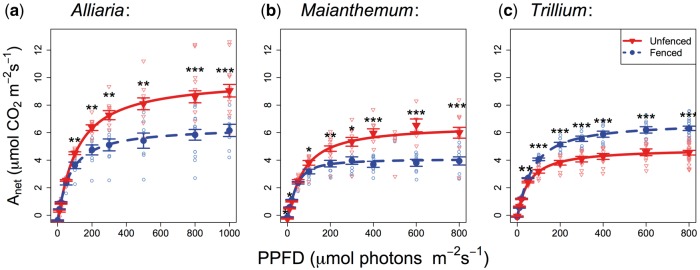
Average mid-season light response curves for (a) *Alliaria petiolata* (rosettes; 19 individuals), (b) *Maianthemum racemosum* (20 individuals) and (c) *Trillium grandiflorum* (19 individuals) in unfenced (red triangles, solid line) and fenced (blue circles, dashed line) areas. Error bars (group mean* *±* *1 SE) indicate empirically measured area-based net photosynthetic rates (*A_net_*) at each irradiance (photosynthetic photon flux density (PPFD)). Statistical differences are between groups evaluated using two-tailed *t*-tests: *P <* *0.05; **P <* *0.01; ***P <* *0.001.

Within species, *A_sat_* did not differ between fenced and unfenced plants during spring months that lacked significant overstory canopy cover (all *P *>* *0.05; Fig. 2). However, all three species exhibited significant treatment differences following overstory canopy closure. *Alliaria* showed a 59 % (*t* = 2.85, df = 218, *P *<* *0.01), 75 % (*t* = 5.85, df = 218, *P *<* *0.001) and 65 % (*t* = 2.81, df = 218, *P *<* *0.01) increase in *A_sat_* in unfenced compared with fenced areas for June, July and August, respectively (Fig. 2A). Similarly, *Maianthemum* in unfenced areas also had higher mean *A_sat_* in June (28 % increase; *t* = 2.87, df = 108, *P *<* *0.01), July (25 % increase; *t* = 2.50, df = 108, *P *<* *0.05) and August (131 % increase; *t* = 2.42, df = 108, *P *<* *0.05; Fig. 2B). By contrast, *Trillium* exhibited the opposite pattern, with a 37 % increase of mean *A_sat_* in unfenced areas, but during May only (*t* = −3.77, df = 73, *P *<* *0.001; Fig. 3C).

#### Midseason light and CO_2_ response curves

Detailed light response curves for *Alliaria* rosettes measured during a single point mid-summer (July) show clear photosynthetic differences between fenced and unfenced areas, with greater gross maximum photosynthetic rates (*A_max_*) in unfenced areas, as well as a significantly greater mean LSP (64 % greater; Fig. 3A, Table 2A). Likewise, both maximum carboxylation capacities (*V_c_*_,__*max*_) and rates of electron transport (*J_max_*) were significantly higher in unfenced versus fenced areas (Table 2A).

**Table 2.** Mean trait values (±1 SE) in fenced and unfenced areas for (a) *Alliaria*, (b) *Maianthemum* and (c) *Trillium*. Statistical differences were determined using likelihood ratio tests (*χ*^2^ with 1 df) that compared models for each trait with and without deer fencing fixed effect (unfenced or fenced) and random effects of individual and/or measurement date.

Trait (units)^a^	Unfenced (deer access)	Fenced (deer exclusion)	Fencing (*χ*^2^)
**(a)***Alliaria petiolata*^b^			
SLA (cm^2^ g^−1^)			
Rosette:	**350*** *** ±*** ***19**	**674*** *** ±*** ***54**	16.85***
Adult:	451* *** ±*** *23	416* *** ±*** *25	1.26
Leaf area (cm^2^)			
Rosette:	**6.21*** *** ±*** ***0.55**	**10.31*** *** ±*** ***1.15**	9.42**
Adult:	21.87* *** ±*** *1.98	19.59* *** ±*** *2.19	1.34
*A_max_* (µmol CO_2_ m^−2^ s^−1^)	**9.40*** *** ±*** ***0.82**	**6.26*** *** ±*** ***0.72**	6.75**
*R_d_* (µmol CO_2_ m^−2^ s^−1^)	0.52* *** ±*** *0.07	0.34* *** ±*** *0.51	2.48
*φ* (µmol CO_2_ µmol^−1^ photons)	0.077* *** ±*** *0.003	0.071* *** ±*** *0.006	0.71
LSP (µmol photons m^−2^ s^−1^)	**643.9*** *** ±*** ***71.6**	**393.3*** *** ±*** ***71.3**	5.45*
LCP (µmol photons m^−2^ s^−1^)	6.9* *** ±*** *0.9	4.7* *** ±*** *0.5	3.33+
*V_c_*_,__*max*_ (µmol CO_2_ m^−2^ s^−1^)	**27.73*** *** ±*** ***1.06**	**11.02*** *** ±*** ***1.09**	37.33***
*J_max_* (µmol e^−^ m^−2^ s^−1^)	**43.49*** *** ±*** ***1.44**	**21.00*** *** ±*** ***1.44**	37.55***
**(b)***Maianthemum racemosum*			
SLA (cm^2^ g^−1^)	**211*** *** ±*** ***6**	**314*** *** ±*** ***29**	9.73**
Leaf area (cm^2^)	**20.74*** *** ±*** ***1.22**	**37.76*** *** ±*** ***4.31**	11.95***
*A_max_* (µmol CO_2_ m^−2^ s^−1^)	**6.66*** *** ±*** ***0.56**	**4.70*** *** ±*** ***0.39**	10.41**
*R_d_* (µmol CO_2_ m^−2^ s^−1^)	**0.24*** *** ±*** ***0.02**	**0.12*** *** ±*** ***0.02**	2.48***
*φ* (µmol CO_2_ µmol^−1^ photons)	**0.075*** *** ±*** ***0.003**	**0.069*** *** ±*** ***0.003**	5.33*
LSP (µmol photons m^−2^ s^−1^)	**468.4*** *** ±*** ***60.3**	**192.4*** *** ±*** ***28.3**	13.40***
LCP (µmol photons m^−2^ s^−1^)	**3.3*** *** ±*** ***0.4**	**1.5*** *** ±*** ***0.2**	16.74***
*V_c_*_,__*max*_ (µmol CO_2_ m^−2^ s^−1^)	24.6* *** ±*** *2.22	22.05* *** ±*** *0.76	0.54
*J_max_* (µmol e^−^ m^−2^ s^−1^)	40.66* *** ±*** *2.61	35.88* *** ±*** *0.68	2.23
**(c)***Trillium grandiflorum*			
SLA (cm^2^ g^−1^)	426* *** ±*** *17	416* *** ±*** *36	0.23
Leaf area (cm^2^)	NA	NA	NA
*A_max_* (µmol CO_2_ m^−2^ s^−1^)	**4.99*** *** ±*** ***0.28**	**6.78*** *** ±*** ***0.19**	12.74***
*R_d_* (µmol CO_2_ m^−2^ s^−1^)	0.13* *** ±*** *0.02	0.19* *** ±*** *0.02	0.19
*φ* (µmol CO_2_ µmol^−1^ photons)	0.089* *** ±*** *0.004	0.084* *** ±*** *0.004	1.08
LSP (µmol photons m^−2^ s^−1^)	451.8* *** ±*** *51.8	512.8* *** ±*** *51.0	1.27
LCP (µmol photons m^−2^ s^−1^)	1.5* *** ±*** *0.3	2.4* *** ±*** *0.2	0.65
*V_c_*_,__*max*_ (µmol CO_2_ m^−2^ s^−1^)	NA	18.89* *** ±*** *1.23	NA
*J_max_* (µmol e^−^ m^−2^ s^−1^)	NA	41.97* *** ±*** *2.38	NA

Statistical significance (*P *<* *0.05) by treatment indicated in bold. +*P *<* *0.1;

* *P *<* *0.05;

** *P *<* *0.01;

*** *P *<* *0.001.

^a^All traits measured in June (*Maianthemum*, *Trillium*) or July (*Alliaria*). SLA, specific leaf area; leaf area, average area of individual leaf (not measured for *Trillium*); *A_max_*, area-based light saturated gross photosynthetic rate; *R_d_*, area-based dark respiration rates at ambient [CO_2_]; *φ*, apparent quantum yield; LSP, 90 % light saturation point; LCP, light compensation point; *V_c_*_,__*max*_, maximum carboxylation rate; *J_max_*, maximum electron transport rate (*Trillium A*/*C_i_* curves for fenced plants only).

^b^Data for rosettes (1st year) unless otherwise indicated.

Mid-season response curves for *Maianthemum* showed qualitatively similar differences between fenced and unfenced areas, as found for *Alliaria* (Fig. 3B, Table 2B). In particular, *A_max_* LSP, LCPs, and apparent quantum yield were greater in unfenced compared with fenced plants. However, *V_c_*_,__*max*_ and *J_max_* did not vary significantly.

Unlike *Maianthemum* and *Alliaria*, *Trillium* exhibited significantly lower *A_max_* in unfenced compared with fenced areas (Fig. 3C) but no other measured parameters differed by treatment (Table 2C).

#### Specific leaf area

Impacts on leaf morphology varied between adult and rosette leaves for *Alliaria*, so we report them separately. Mean specific leaf area (SLA) for rosette leaves was significantly higher in fenced compared with unfenced areas, and these leaves were also significantly larger (Table 2A). However, *Alliaria* adults did not differ in SLA or leaf size between fenced and unfenced areas. Like *Alliaria* rosettes, *Maianthemum* had greater SLA and larger leaves on average in fenced areas (Table 2B). By contrast, SLA did not differ for *Trillium* (Table 2C).

## Discussion

Despite the growing body of evidence that invasion can be strongly modulated by overabundant ungulate herbivores (Vavra *et al.* 2007; Eschtruth and Battles 2009; Knight *et al.* 2009; Kalisz *et al.* 2014; Christopher *et al.* 2014; Dávalos *et al.* 2015*a*; Shen *et al.* 2016), many studies on the functional ecology of plant invasions do not consider these effects. Here, we investigated the potential physiological consequences of deer exclusion on *Alliaria*. We found pronounced differences in the photosynthetic performance of this invader between fenced and unfenced areas. Notably, during summer months, *Alliaria* had a ∼40 % reduction in mean light-saturated photosynthetic rates (*A_sat_*) and 93 % increase in SLA when deer were excluded. Reduced *A_sat_* and increased SLA are both known responses to shade (Valladares and Niinemets 2008). The palatable native perennials differed in response to deer exclusion: *Maianthemum* showed deer-mediated photosynthetic increases while *Trillium* exhibited nearly the opposite pattern. While our data suggest the importance of deer on invasive plant performance, we did not measure additional factors that might also contribute to these responses, including earthworm invasion (Nuzzo *et al.* 2009) and the interaction between earthworms, deer and plant invasions (Dávalos *et al.* 2015*a*, b).

Why did *Alliaria* exhibit strong physiological advantages due to overabundant deer? In line with our predictions, *Alliaria* photosynthetic rates were congruent with previous demographic results from the study site, where deer not only facilitate, but *enable*, positive population growth rate (Kalisz *et al.* 2014). Knight *et al.* (2009) reported relatively greater area unoccupied by herbaceous vegetation in unfenced (deer access) plots, which suggested declines in the native understory canopy and presumably higher understory light levels. The current study quantitatively confirmed this idea, with a nearly threefold mean increase in leaf-level light availability where deer were present. We propose that these deer-associated differences in understory light availability are due to high browse pressure on palatable natives (Kalisz *et al.* 2014), as well indirect effects on other less preferred plant species in the community (Rooney and Waller 2003; Heckel *et al.* 2010).

The relative importance of direct and indirect deer effects versus other factors remains unknown. Additionally, we only measured instantaneous PPFD at the leaf-level of each target plant, so the relative contributions by the overstory, mid-story, shrub or herb layer to these differences in light availability remain unclear. Given deer have been excluded for only 15 years at this site and the browse line is typically <2 m, we assume the overstory canopy is similar in fenced and unfenced areas where we took our measurements. Further research is needed to confirm this assumption at this site. In a study of 44 exclosures in the eastern US, Rohleder (2013) found no difference in overstory canopy cover between fenced and unfenced plots, but the density of vegetation <2 m was significantly greater in fenced plots.

*Alliaria*’s trait responses to deer exclusion closely matched expectations from the ecophysiological literature on trait responses and adaptations to shade (Valladares and Niinemets 2008). First, decreased area-based photosynthetic rates (*A_max_*, *A_net_*), carboxylation rates (*V_c_*_,__*max*_) and electron transport rates (*J_max_*), as we found for *Alliairia* in fenced areas during summer, are characteristic of shade tolerant species (Valladares and Niinemets 2008) and within species, individuals acclimated to low light conditions (Evans and Poorter 2001). Further, both SLA and leaf area were greater in fenced areas, as expected for forest herbs under low light levels (Rothstein and Zak 2001). In both shade tolerant and intolerant species, increasing SLA is a common response to low light, which is thought to maximize carbon gain per unit mass by increasing light capture area (Evans and Poorter 2001). We did not measure soil nutrients or leaf chemistry (e.g. leaf N) and these factors could potentially differ between fenced and unfenced areas. Collectively, *Alliaria*’s traits in fenced areas are indicative of a shift towards a more shade tolerant strategy, although its growth and reproduction are substantially reduced in low light environments (Stinson and Seidler 2014). Trait differences between fenced and unfenced areas are likely due to acclimation to growth conditions (phenotypic plasticity) rather than genetic changes. However, if deer-mediated selection pressures remain strong, there is potential for evolutionary change (Heckel and Kalisz 2017).

Light availability is often the dominant limiting factor for plant growth in the forest understory (Finzi and Canham 2000) and the main resource axis that differentiates ecophysiologicial strategies of forest herbs (Neufeld and Young 2014). The abundance of invasive species in forested habitats has been empirically linked to higher light environments (e.g. Dreiss and Volin 2013). While shade tolerant strategies can permit invasion and persistence in forest understories (Martin *et al.* 2009), both natives and non-native invaders in the understory can benefit from increased light (Heberling and Fridley 2016). *Alliaria* has highest fitness in forest edge habitats but can survive and reproduce in a variety of light environments (Meekins and Mccarthy 2001). Its invasion is particularly puzzling as it can invade seemingly intact forest understories (Nuzzo 1999; Rodgers et al. 2008) where light availability is relatively low. We suggest that overabundant deer create light conditions in the forest interior similar to edge habitats. Indeed, previous studies report positive correlations between *Alliaria* photosynthetic rates and irradiance (Dhillion and Anderson 1999; Myers and Anderson 2003; Myers *et al.* 2005). One hypothesis for *Alliaria’s* success focuses on its ‘extended’ phenology as a biennial, compared with the native species in communities it invades (Engelhardt and Anderson 2011; Smith and Reynolds 2015). This ‘extended phenology’ hypothesis emphasizes the importance of *Alliaria*’s ability to capitalize early- and late-season light levels for invasion success. Further, Stinson and Seidler (2014) found *Alliaria* growing in forest edge habitat conditions achieve higher photosynthetic rates, lower SLA, and higher fecundity than those in the forest interior (low light). In a common garden, they found that these physiological differences between forest edge and interior populations are due to plasticity and conclude that genetic adaptation to shade in this species might be constrained by trade offs with plastic trait responses that optimize fitness at higher light availabilities. Our results broaden these prior findings to connect light-associated advantages for *Alliaria*’s physiology to deer overabundance, a phenomenon that is nearly ubiquitous across its invaded range (McShea 2012) yet only briefly mentioned in a major review of factors driving *Alliaria*’s invasive success (Rodgers *et al.* 2008).

Similar to responses in *Alliaria*, the native perennial *Maianthemum* exhibited higher photosynthetic performance and lower SLA compared with fenced plants. These functional shifts toward a high light-adapted plant strategy are expected when considering light-level differences, as this result is not due to deer *per se* but rather deer-mediated indirect effects via differences in light availability. The contrasting result seen in *Trillium*, which displayed *lower* photosynthetic rates in June in unfenced compared with fenced areas, is surprising. As both *Trillium* and *Maianthemum* are presumably light-limited under closed canopies (Neufeld and Young 2014), both species should benefit from increased understory light availability. However, June leaf-level PPFD data taken concurrently with gas-exchange data for *Trillium* indicated the opposite pattern, with PPFD slightly lower in unfenced areas (mean June PPFD ± SE: 34 ± 4 μmol photons m^−2^ s^−1^ vs. 23 ± 8 μmol photons  m^−2^ s^−1^,  for fenced vs. unfenced areas, respectively). (Note: *Trillium* was the only species measured in June 2016, so these PPFD data should be interpreted with caution). One explanation for the decrease in photosynthetic rates of *Trillium* in the presence of deer could be that the species is less phenotypically plastic with respect to its leaf physiology than *Maianthemum*, as suggested by its slightly earlier spring emergence, mid-summer senescence and lack of SLA signal. As a result, C gain in late spring, after canopy closure when understory light levels substantially decrease, might be constrained such that light availability is not the factor explaining the difference in photosynthetic rates between fenced and unfenced areas. Additionally, *Trillium* is a highly preferred forage of deer and may be more likely to be browsed when growing in microsites with higher light availability than *Maianthemum*. Therefore, our sampling of *Trillium* in unfenced areas may have overrepresented relatively shadier microsites despite deer access. Another possibility is that *Trillium* recruitment was biased toward high light microsites where deer have access. More data are required to fully address these hypotheses, including measurements of plant size, age, and herbivory and flowering history. Together, our data suggest that while deer exclusion influence plant ecophysiology, the response can differ by species.

Previous research has strongly linked deer to *Alliaria* invasions (e.g. Kalisz *et al.* 2014), but does the species’ widespread invasive success *require* deer? There are many additional factors that are increasingly appreciated as causing ecosystem modification that can affect invasion success, including invasive earthworms (e.g. Dávalos *et al.* 2015a). Invasive earthworms are ubiquitous at our study site and have been found to play an important role in facilitating plant invasion (Nuzzo et al. 2009). Further, it is important to note that our study does not provide direct, causal evidence that deer are solely responsible for increases in light availability and the ecophysiological differences we report, or that light availability alone fully explains differences in photosynthetic rates between fenced and unfenced areas. Additionally, the intensity and ecological importance of species’ responses to deer exclusion will likely differ based on co-varying, idiosyncratic factors such as community context, competition, forest successional stage, local deer abundance, landscape conditions and disturbance history. However, in combination with demographic models and population-level studies (Eschtruth and Battles 2009; Kalisz *et al.* 2014), our new results highlight the potential importance of deer for plant invasion. At our study site, deer were only recently overabundant (since ∼1992), and *Alliaria* was rare prior to this deer overabundance (Knight *et al.* 2009). Interestingly, an herbarium specimen (Henry s.n. [CM 046953]) documents that *Alliaria* was collected at Trillium Trail in 1956, nearly 40 years prior to the species becoming locally invasive. While not conclusive, these separate lines of evidence (demographic, physiological, historical record) collectively support the hypothesis that the species’ invasion is strongly dependent upon habitat modifications by overabundant deer. While it is unknown the degree to which our conclusions can be generalized to other invasive taxa or ecosystems, overabundant ungulates may play an important role in facilitating some plant invasions (Shen *et al.* 2016; Averill *et al.* this issue).

## Conclusions

Ecosystems subjected to frequent disturbance have long been thought to be more susceptible to invasion (Elton 1958; Hobbs and Huenneke 1992). However, seemingly undisturbed forests are not immune to invasion, especially by shade-tolerant non-native species (Martin *et al.* 2009), and all forests are subject to anthropogenic disturbance to some degree. Modern forests are commonly subject to multiple ecological stressors (Dávalos *et al.*2015*a*), including modifications to the abiotic and biotic environment modulated by deer overabundance. In the present study, we found the presence of deer can modify seasonal light availability in the understory herb layer. As a result, invasive *Alliaria* expressed a sun-adapted physiological strategy when deer were present. Although deer-mediated facilitation in *Alliaria* supports the ‘passengers’ model of species invasions (MacDougall and Turkington 2005), previous studies at the same site show that, even when deer are present, *Alliaria* removal can significantly benefit native plant vital rates (Brouwer *et al.* 2015).

Overabundant ungulates act as a strong biotic force that result in significant interspecific shifts in phylogenetic diversity (Begley-Miller *et al.* 2014) and community-weighted functional trait composition (Mason *et al.* 2010). Little research has been published on intraspecific ecophysiological differences mediated by generalist mammalian herbivores. In line with previous studies (e.g. Knight *et al.* 2009; Kalisz *et al.* 2014), this link between deer presence and individual plant performance highlights the need for management of deer to control invasions, particularly by plant species requiring higher light, openings in the understory canopy or disturbance.

## Sources of Funding

This work was supported by the US National Science Foundation (DEB 1457531, DEB 144552, DEB 0958676) and the University of Tennessee Knoxville.

## Contribution by the Authors

J.M.H. and S.K. conceived the study, J.M.H. performed field work and measurements, N.L.B. and J.M.H. analysed data, J.M.H. led the writing with revisions from all authors.

## Conflicts of Interest Statement

No conflicts of interest.

## Supplementary Material

Supplementary Data

## Supporting Information

The following additional information is available in the online version of this article—

**File S1.** Annotated R code used for fitting photosynthetic models to response curve data.

**Table S1.** Light response curve summary data.

**Table S2.** *A*/*C_i_* curve summary data.

**Table S3.** SLA data.

**Figure S1.** Understory light level data used to estimate date of overstory canopy closure.

**Figure S2.** Derivative approach to estimate date of spring overstory canopy closure.
