# Supplementary material for: Effects of deer on the photosynthetic performance of invasive and native forest herbs
Source: AoB Plants. 2017 Mar 20;9(2):plx011. doi: 10.1093/aobpla/plx011 (PMC5424084; doi:10.1093/aobpla/plx011)
Supplement: Supplementary Data [file plx011_Supp.zip › Supporting information.docx]

Heberling JM, Brouwer NL, Kalisz S. 2017. Effects of deer on the photosynthetic performance of invasive and native forest herbs. *AoB PLANTS.*

**SUPPORTING INFORMATION**

**Fig. S1**. Understory light levels during and after canopy closure in the large deer exclosure at Trillium Trail, Fox Chapel, PA. PPFD (photosynthetic photon flux density; μmol photons m^-2^ s^-1^) was measured at 1.5m height using a quantum sensor (Onset Computer Corp, Bourne, MA, USA) from 15 April 2016 - 07 July 2016. PPFD was measured every minute and 30 min averages were logged. Data points represent the overall mean of half-hour measurement averages from 1000 h to 1400 h. Error bars show 95% CIs. Blue line shows GAM smoother with SE shown in grey. Note: the large error bar for the first date is because the logger was deployed mid-day, resulting in fewer logged points than all other dates.

**Figure S2.** Derivative of GAM model for midday understory light levels (photosynthetic photon flux density; PPFD; μmol photons m^-2^ s^-1^) through time. Dashed lines show 95% CIs. When derivative equals zero (red solid line), PPFD levels remain constant (i.e., overstory canopy closure completed). Vertical bars indicate point where 95% CI and point estimate of derivative first cross zero.

**File S1.** Annotated R code for fitting photosynthetic models to response curve data.

# Sample code for fitting CO2 (A/Ci) and light (A/q) response curves using nonlinear least squares approach

# JM Heberling (jmheberling@gmail.com)

# Sample data and full annotated code posted here:

#https://sites.google.com/site/fridleylab/home/protocols

# *************

#Note that errors from R are most likely due to convergence issues that stem from too little data or starting values.

# *************

# See Heberling & Fridley (2016) Ecology for code for Bayesian estimation of Farquar et al. (1980) model, with references therein.

#===================================================

# Modeling light response curves (aka A/Q or LRC)

# Model of Marshall & Biscoe (1980) J Exp Bot 31:29-39 and elsewhere

#===================================================

 # Read in text file from Licor 6400

lrc<- read.csv("/YOURDIRECTORYHERE/sample_lrc.txt",sep="",skip=16) #based on standard LI-6400 text file outputs

# ---Inspect and graph raw data (A vs. PPFD) ---

PARlrc<-lrc$PARi #PAR (aka PPFD or Q)

photolrc<-lrc$Photo #net photosynthetic rate (Anet)

curvelrc<-data.frame(PARlrc,photolrc)

curvelrc

par(mar=c(3,3,0,0),oma=c(1.5,1.5,1,1))

plot(PARlrc,photolrc,xlab="", ylab="", ylim=c(-2,max(photolrc)+2),cex.lab=1.2,cex.axis=1.5,cex=2)

mtext(expression("PPFD ("*mu*"mol photons "*m^-2*s^-1*")"),side=1,line=3.3,cex=1.5)

mtext(expression(A[net]*" ("*mu*"mol "*CO[2]*" "*m^-2*s^-1*")"),side=2,line=2.5,cex=1.5)

# --- Nonlinear least squares regression (non-rectangular hyperbola).  4 parameter model: Amax (max gross photosytnthetic rate), Rd (dark respiration), AQY (apparent quantum yield), Theta (curvature parameter, dimensionless) ---

curve.nlslrc = nls(photolrc ~ (1/(2*theta))*(AQY*PARlrc+Am-sqrt((AQY*PARlrc+Am)^2-4*AQY*theta*Am*PARlrc))-Rd,start=list(Am=(max(photolrc)-min(photolrc)),AQY=0.05,Rd=-min(photolrc),theta=1))

summary(curve.nlslrc) #summary of model fit

# ---Graph raw data with modeled curve---

par(mar=c(3,3,0,0),oma=c(1.5,1.5,1,1))

plot(PARlrc,photolrc,xlab="", ylab="", ylim=c(-2,max(photolrc)+2),cex.lab=1.2,cex.axis=1.5,cex=2)

mtext(expression("PPFD ("*mu*"mol photons "*m^-2*s^-1*")"),side=1,line=3.3,cex=2)

mtext(expression(A[net]*" ("*mu*"mol "*CO[2]*" "*m^-2*s^-1*")"),side=2,line=2,cex=2)

curve((1/(2*summary(curve.nlslrc)$coef[4,1]))*(summary(curve.nlslrc)$coef[2,1]*x+summary(curve.nlslrc)$coef[1,1]-sqrt((summary(curve.nlslrc)$coef[2,1]*x+summary(curve.nlslrc)$coef[1,1])^2-4*summary(curve.nlslrc)$coef[2,1]*summary(curve.nlslrc)$coef[4,1]*summary(curve.nlslrc)$coef[1,1]*x))-summary(curve.nlslrc)$coef[3,1],lwd=2,col="blue",add=T)

# ---Solve for light compensation point (LCPT), PPFD where Anet=0 ---

x<-function(x) {(1/(2*summary(curve.nlslrc)$coef[4,1]))*(summary(curve.nlslrc)$coef[2,1]*x+summary(curve.nlslrc)$coef[1,1]-sqrt((summary(curve.nlslrc)$coef[2,1]*x+summary(curve.nlslrc)$coef[1,1])^2-4*summary(curve.nlslrc)$coef[2,1]*summary(curve.nlslrc)$coef[4,1]*summary(curve.nlslrc)$coef[1,1]*x))-summary(curve.nlslrc)$coef[3,1]}

uniroot(x,c(0,50))$root #LCPT

# ---Solve for light saturation point (LSP), PPFD where 75% of Amax is achieved (75% is arbitrary - cutoff could be changed)

x<-function(x) {(1/(2*summary(curve.nlslrc)$coef[4,1]))*(summary(curve.nlslrc)$coef[2,1]*x+summary(curve.nlslrc)$coef[1,1]-sqrt((summary(curve.nlslrc)$coef[2,1]*x+summary(curve.nlslrc)$coef[1,1])^2-4*summary(curve.nlslrc)$coef[2,1]*summary(curve.nlslrc)$coef[4,1]*summary(curve.nlslrc)$coef[1,1]*x))-summary(curve.nlslrc)$coef[3,1]-(0.75*summary(curve.nlslrc)$coef[1,1])+0.75*(summary(curve.nlslrc)$coef[3,1])}

uniroot(x,c(0,1000))$root #LSP

#===================================================

# Modeling CO2 response curves (A/Ci)

# Farquhar-von Caemmerer-Berry (FvCB) model (1980) Planta 149:78-90

# Simultaneous estimation detailed in Dubois et al. (2007) New Phyt 176:402-414

#===================================================

 # Read in text file from Licor 6400

aci<- read.csv("/YOURDIRECTORYHERE/sample_aci.txt", sep="",skip=16)

aci<-na.omit(aci) #remove lines with remarks (*check notebook/file for comments)

# ---Inspect and graph raw data (A vs. Ci) ---

Ci<-aci$Ci # Ci (ppm)

Ci_Pa<-aci$Ci_Pa # Ci (Pa)

Photo<-aci$Photo #Anet - net photosynthetic rate

CO2R<-aci$CO2R

TotalCurveData<-data.frame(Ci, CO2R, Photo, Ci_Pa)

TotalCurveData # *inspect raw data and check notebook (data reasonable or need edited/discarded?)

par(mar=c(3,3,0,0),oma=c(1.5,1.5,1,1))

plot(Ci_Pa,Photo,ylab="", xlab="",cex.lab=1.2,cex.axis=1.5,cex=2)

mtext(expression("Intercellular "*CO[2]*" Pressure (Pa)"),side=1,line=3.3,cex=1.5)

mtext(expression(A[net]*" ("*mu*"mol "*CO[2]*" "*m^-2*s^-1*")"),side=2,line=2.5,cex=1.5)

# ---Temperature adjusted coefficients:

# Constants published in Sharkey et al (2007) Plant Cell Env 30: 1035-1040

R=0.008314 #(kJ mol^-1 K^-1)

aci$Kc=exp(35.9774-80.99/(R*(aci$Tleaf+273.15))) #Michaelis-Menten constant for Rubisco for O2 (Pa)

aci$Ko=exp(12.3772-23.72/(R*(aci$Tleaf+273.15))) #Michaelis-Menten constant for Rubisco for CO2 (kPa)

aci$GammaStar=exp(11.187-24.46/(R*(aci$Tleaf+273.15))) #Photorespiration compensation point (Pa)

O=21 #oxygen (O2) partial pressure (kPa)

# ---RUPB saturated portion---

#(Vcmax*(Ci_Pa-GammaStar))/(Ci_Pa+(Kc*(1+(O/Ko)))))-Rd

# ---RUBP limited portion---

#((J*(Ci_Pa-GammaStar))/((4*Ci_Pa)+(8*GammaStar)))-Rd

aci.fit<-nls(Photo~ifelse(((Vcmax*(Ci_Pa-GammaStar))/(Ci_Pa+(Kc*(1+(O/Ko)))))<((J*(Ci_Pa-GammaStar))/((4*Ci_Pa)+(8*GammaStar))),((Vcmax*(Ci_Pa-GammaStar))/(Ci_Pa+(Kc*(1+(O/Ko))))),((J*(Ci_Pa-GammaStar))/((4*Ci_Pa)+(8*GammaStar))))-Rd,start=list(Vcmax=50,J=100,Rd=0.5),data=aci) #if error: reconsider starting values, but most likely a dataset issue. (too few points or response curve not clear; fail to reach solution)

summary(aci.fit)

Vcmax<-summary(aci.fit)$coef[1,1]

J<-summary(aci.fit)$coef[2,1]

Rd<-summary(aci.fit)$coef[3,1]

# ---Graph raw data with modeled curve---

par(mar=c(3,3,0,0),oma=c(1.5,1.5,1,1))

plot(Ci_Pa,Photo,ylab="", xlab="",cex.lab=1.2,cex.axis=1.5,cex=2)

mtext(expression("Intercellular "*CO[2]*" Pressure (Pa)"),side=1,line=3.3,cex=1.5)

mtext(expression("Net photosynthetic rate  (umol  "* CO[2]*   m^-2*   s^-1*")"),side=2,line=2.5,cex=1.5)

curve(ifelse(((Vcmax*(x-mean(aci$GammaStar)))/(x+(mean(aci$Kc)*(1+(O/mean(aci$Ko))))))<((J*(x-mean(aci$GammaStar)))/((4*x)+(8*mean(aci$GammaStar)))),((Vcmax*(x-mean(aci$GammaStar)))/(x+(mean(aci$Kc)*(1+(O/mean(aci$Ko)))))),((J*(x-mean(aci$GammaStar)))/((4*x)+(8*mean(aci$GammaStar)))))-Rd,add=T) #Reasonable fit? Could check goodness of fit, model assumptions
